# Comparison of transcatheter edge-to-edge and surgical repair in patients with functional mitral regurgitation using a meta-analytic approach

**DOI:** 10.3389/fcvm.2022.1063070

**Published:** 2023-01-25

**Authors:** D. Felbel, M. Paukovitsch, R. Förg, T. Stephan, B. Mayer, M. Keßler, M. Tadic, T. Dahme, W. Rottbauer, S. Markovic, L. Schneider

**Affiliations:** ^1^Department of Cardiology, Angiology, Pneumology and Intensive Care, University Hospital Ulm, Ulm, Germany; ^2^Institute of Epidemiology and Medical Biometry, Ulm University, Ulm, Germany

**Keywords:** functional mitral regurgitation, transcatheter edge-to-edge repair, surgical mitral valve repair, heart failure, mitraclip

## Abstract

**Background:**

Evidence regarding favorable treatment of patients with functional mitral regurgitation (FMR) using transcatheter edge-to-edge repair (TEER) is constantly growing. However, there is only few data directly comparing TEER and surgical mitral valve repair (SMVr).

**Aims:**

To compare baseline characteristics, short-term and 1-year outcomes in FMR patients undergoing mitral valve (MV) TEER or SMVr using a meta-analytic approach.

**Methods:**

Systematic database search identified 1,703 studies reporting on TEER or SMVr for treatment of FMR between January 2010 and December 2020. A meta-analytic approach was used to compare outcomes from single-arm and randomized studies based on measures by means of their corresponding 95% confidence intervals (CI). Statistical significance was assumed if CIs did not overlap. A total of 21 TEER and 37 SMVr studies comprising 4,304 and 3,983 patients were included.

**Results:**

Patients in the TEER cohort presented with higher age (72.0 ± 1.7 vs. 64.7 ± 4.7 years, *p* < 0.001), greater burden of comorbidities like hypertension (*p* < 0.001), atrial fibrillation (*p* < 0.001), lung disease (*p* < 0.001) and chronic renal disease (*p* = 0.005) as well as poorer left ventricular ejection fraction (30.9 ± 5.7 vs. 36.6 ± 5.3%, *p* < 0.001). In-hospital mortality was significantly lower with TEER [3% (95%-CI 0.02–0.03) vs. 5% (95%-CI 0.04–0.07)] and 1-year mortality did not differ significantly [18% (95%-CI 0.15–0.21) vs. 11% (0.07–0.18)]. NYHA [1.06 (95%-CI 0.87–1.26) vs. 1.15 (0.74–1.56)] and MR reduction [1.74 (95%-CI 1.52–1.97) vs. 2.08 (1.57–2.59)] were comparable between both cohorts.

**Conclusion:**

Despite considerably higher age and comorbidity burden, in-hospital mortality was significantly lower in FMR patients treated with TEER, whereas a tendency toward increased 1-year mortality was observed in this high-risk population. In terms of functional status and MR grade reduction, comparable 1-year results were achieved.

## Introduction

Over the past decade transcatheter edge-to-edge repair (TEER) using the MitraClip^®^ (MC) system (Abbott Vascular, Santa Clara, CA, USA) emerged as an important treatment option for mitral regurgitation (MR). In the initial EVEREST trials the MC was compared to surgical mitral valve repair (SMVr) mainly in patients suffering from degenerative MR (DMR) (1, 2). However, following its broad and successful implementation in Europe, the MC received CE mark approval for both etiologies in 2008. Since then, data regarding patients with functional MR (FMR) included in the EVEREST trials (1, 2), REALISM (1), TVT registries (3) as well as European registries like ACCESS EU (4), GRASP (5), TRAMI (6), and Sentinel (7) demonstrated device safety and adequate MR reduction with the MC. After FDA approval for DMR in 2013, the randomized COAPT trial confirmed the efficacy of the MC system in patients with FMR compared to medical treatment (8). Simultaneously, the European MITRA-FR study, a second randomized trial comparing TEER and optimal medical therapy, failed to show benefits of MC therapy in FMR patients (9). However, these diametrically opposed results were attributed to differences in patient selection and trial design (10). Accordingly, the MC received FDA approval for FMR in 2019 and novel ACC guidelines recommend TEER in FMR patients with adequate anatomy and left ventricular ejection fraction (LVEF), whereas ESC guidelines reserve TEER for FMR patients not eligible for surgery (11, 12). Recently, the PASCAL^®^ (Edwards Lifesciences, Irvine, CA, USA) was introduced as a second TEER system providing sparse but promising data so far (13). However, there still is only few data directly comparing TEER and SMVr in patients with FMR and due to limited studies (14) reporting on FMR only, previous meta-analyses (15, 16) comparing TEER and SMVr included both FMR and DMR making interpretation difficult (17).

In this study, we gathered available data and compared results and outcomes of TEER using the established MC and SMVr for FMR exclusively by using a meta-analytic approach.

## Materials and methods

### Search strategy and study selection

A systematic database search was performed in MEDLINE and Embase for studies published from January 2010 to December 2020 reporting on TEER or SMVr for treatment of FMR. Due to limited data regarding use of the novel PASCAL^®^ system in FMR patients and its early implementation, this investigation focused on the well-established MC. MeSH terms included “mitral valve insufficiency,” “ventricular dysfunction,” “cardiac surgical procedures,” and “secondary mitral regurgitation” identifying 1,703 studies. Further details about the search strategy can be found in Supplementary Figure 1.

**FIGURE 1:**
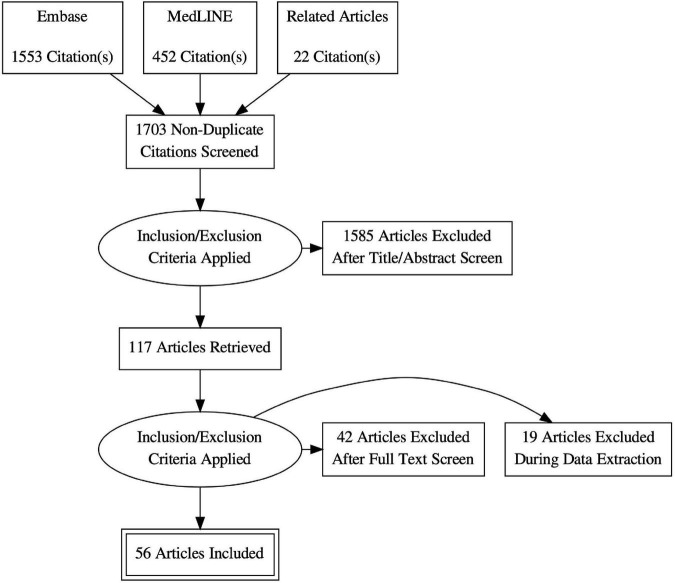
Flow chart of literature search. Created with: PRISMA flow diagram generator.

We included studies reporting in-hospital, 30-day or 1-year death figures for MC or SMVr. Studies reporting data of patients with DMR and FMR were only included if event numbers were quoted separately for each etiology. The flow chart of literature search is presented in Figure 1.

All included studies used MR grading 1–4 or mild, moderate, moderate-to severe and severe, which were assessed by contributed echocardiographers according to current European or American guidelines, vena contracta, jet size or eyeballing. Few studies did not explicitly report grading criteria.

### Data extraction

Two investigators (DF and RF) independently reviewed all articles, selected eligible studies and collected data of interest. Baseline characteristics such as age, gender, LVEF, EuroSCORE, and clinical outcomes (in-hospital, 30-day, 1-year mortality and reoperation or reintervention) were extracted from each study, if available. A third investigator (LS) reviewed differences between the collected data.

### Statistical analysis

Available baseline characteristics are displayed as weighted percentage means with standard deviation for dichotomous variables and weighted means with standard deviation for continuous variables. Categorial variables were treated as quasi-continuous by percentages ranging between 0 and 1 considering a different size of cohorts and studies reporting each variable and treatment. The number of studies reporting the analyzed variable is displayed in Table 1. Mean with standard deviation was calculated using Microsoft Excel (Version 16) for each cohort and compared with the unpaired *t*-test using the *t*-test Calculator by GraphPad online.

**TABLE 1:** Baseline characteristics of patients in the TEER and SMVr cohort.

	TEER 4,304 patients (21 studies)	SMVr 3,983 patients (37 studies)	*P*-value
Age, years	72.0 ± 1.7 (21)	64.7 ± 4.7 (36)	**<0**.**001**
Male%	70.7 ± 7.3 (21)	67.8% ± 9.9 (36)	0.24
Hypertension%	74.5 ± 9.8 (15)	58.9 ± 12.8 (20)	**<0**.**001**
Atrial fibrillation%	51.6 ± 13.4 (18)	26.0 ± 14.8 (28)	**<0**.**001**
Prior MI%	40.0 ± 15.0 (12)	28.7 ± 32.4 (9)	0.30
Prior PCI%	40.7 ± 12.1 (15)	28.8 ± 16.9 (11)	**0**.**048**
Prior CABG%	37.9 ± 10.7 (15)	9.1 ± 8.2 (10)	**<0**.**001**
NYHA III/IV%	65.1 ± 7.6 (20)	60.4 ± 16.3 (20)	0.24
NYHA IV%	20.2 ± 9.7 (19)	20.1 ± 17.5 (8)	0.99
Chronic renal disease%	33.8 ± 14.3 (9)	16.8 ± 11.1 (13)	**0**.**005**
Diabetes%	37.1 ± 6.0 (17)	14.8 ± 47.9 (32)	0.06
Prior stroke%	10.6 ± 3.7 (6)	9.5 ± 4.4 (10)	0.63
EuroSCORE II	15.3 ± 9.0 (6)	11.1 ± 4.1 (5)	0.37
Logistic EuroSCORE	22.4 ± 4.4 (11)	9.1 ± 3.2 (6)	**<0**.**001**
EuroSCORE	22.0 ± 23.9 (2)	9.9 ± 3.3 (5)	0.25
LVEF%	30.9 ± 5.7 (21)	36.6 ± 5.3 (35)	**<0**.**001**
MR grade	3.4 ± 0.4 (15)	3.3 ± 0.5 (21)	0.70
COPD/lung disease%	23.2 ± 5.5 (15)	12.0 ± 5.9 (13)	**<0**.**001**
TR ≥ 2%	46.2 ± 12.5 (5)	30.6 ± 26.8 (2)	0.31

Continuous variables are displayed as mean ± standard deviation; the number of studies reporting the variable by counts are stated in parenthesis. CABG, coronary artery bypass graft; COPD, chronic obstructive pulmonary disease; LVEF, left ventricular ejection fraction; MI, myocardial infarction; MR, mitral regurgitation; PCI, percutaneous coronary intervention; NYHA, New York heart association class; SMVr, surgical mitral valve repair; TEER, transcatheter edge-to-edge repair; TR, tricuspid regurgitation. Significant *p*-values are presented in bold.

### Meta-analytic approach

Since only few studies were available that enabled a direct comparison of TEER and SMVr, a classical meta-analysis was not feasible. In order to combine results from all identified studies regarding in-hospital, 30-day and 1-year mortality as well as reoperation and reintervention rates, New York Heart Association (NYHA) class and MR grade reduction, a meta-analytic approach was used. In particular, the reported overall proportions from the included single-arm studies were combined using the inverse variance method, which is available in the R package “meta”. Study heterogeneity was assessed using the *I*^2^ measure leading to a fixed effects combination model in case of *I*^2^ < 50 and to a random effects combination model otherwise. Furthermore, meta-regression (R package “metafor”) was applied in order to account for possible confounding of the results by different patient characteristics. For a statistical comparison of the overall proportions calculated for each procedure separately, the 95% confidence intervals (CI) were used, where non-overlapping CIs indicated statistical significance (*p* < 0.05) (18).

The study quality was assessed as described in detail by the National Institutes of Health Quality Assessment Tool. Studies were rated as being of either “good”, “fair” or “poor” quality (19). Statistical supervision was provided by the Institute of Epidemiology and Medical Biometry of Ulm University.

All analyses were based on previous studies, therefore neither patient consent nor ethical committee approval was required for this analysis. The investigation conforms with the principles outlined in the Declaration of Helsinki.

## Results

**FIGURE 2:**
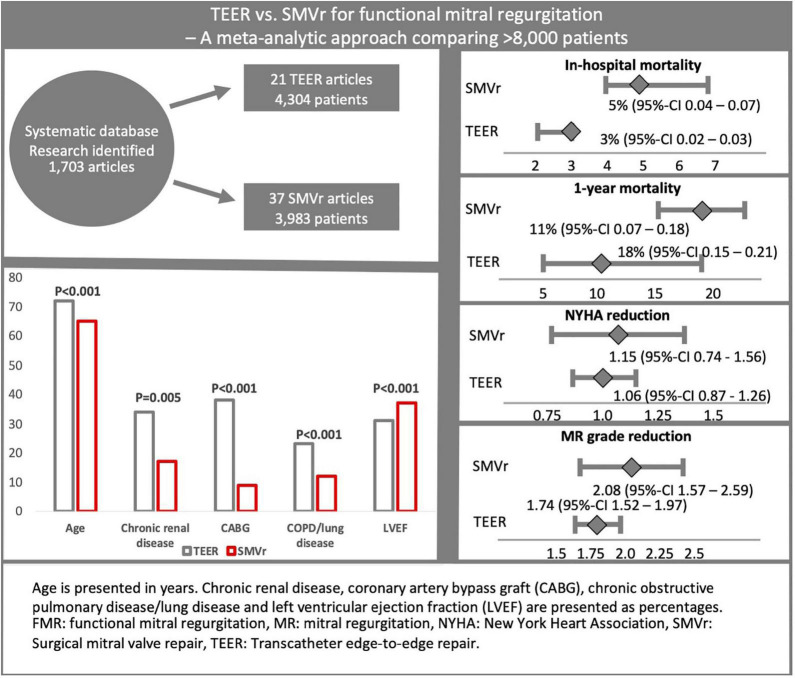
This graphical abstract summarizes study design, the most important baseline characteristics as well as key results of this meta-analytic approach comparing TEER and SMVr in FMR patients. Following systematic database search, 21 TEER and 37 SMVr studies comprising >8,000 patients were included. Despite a considerably higher age and comorbidity burden, in-hospital mortality was significantly lower in the TEER cohort. NYHA and MR reduction were comparable between both treatment strategies.

Literature search identified 1,703 articles. After further analysis 56 studies were considered eligible for inclusion (Figure 1). A total of 21 studies reported data of 4,304 patients treated with TEER, whereas 37 studies reported data of 3,983 patients treated with SMVr (see also Figure 2).

### Repair techniques

The included surgical studies mainly used annuloplasty for MV repair (20–22). Several studies additionally reported chordal cutting (23), chordal replacement (21), papillary muscle relocation (20, 24) or left ventricular remodeling (24, 25). Only one study explicitly reported edge-to-edge repair in combination with annuloplasty (26). Regarding TEER, all included studies used first and second generation MC exclusively.

### Baseline characteristics

Patients in the TEER cohort were significantly older (71.9 ± 1.7 vs. 64.7 ± 4.7 years, *p* < 0.001) and presented significantly more often with hypertension (74.5 ± 9.8 vs. 58.9 ± 12.8%, *p* < 0.001), atrial fibrillation (51.6 ± 13.4 vs. 25.9 ± 14.8%, *p* < 0.001), history of coronary artery bypass graft (37.9 ± 10.7 vs. 9.1 ± 8.2%, *p* < 0.001), chronic obstructive pulmonary disease or lung disorder (23.2 ± 5.5 vs. 12.0 ± 5.9, *p* < 0.001), previous percutaneous coronary intervention (40.7 ± 12.1 vs. 28.8 ± 16.9%, *p* = 0.048), and chronic renal disease (33.8 ± 14.3 vs. 16.8 ± 11.1%; *p* = 0.005). Moreover, patients in the TEER cohort showed poorer LVEF (30.9 ± 5.7 vs. 36.6 ± 5.3%, *p* < 0.001; see Figure 2). Accordingly, logistic EuroSCORE was significantly higher in the TEER cohort (22.4 ± 4.4 vs. 9.1 ± 3.2, *p* < 0.001; see Table 1). No significant differences were observed regarding the rate of prior myocardial infarction (*p* = 0.29) as well as NYHA class (*p* = 0.24), diabetes (*p* = 0.06), severity of tricuspid regurgitation ≥ grade 2 (*p* = 0.31), and MR grade (*p* = 0.70; see also Table 1).

### Short term mortality

**FIGURE 3:**
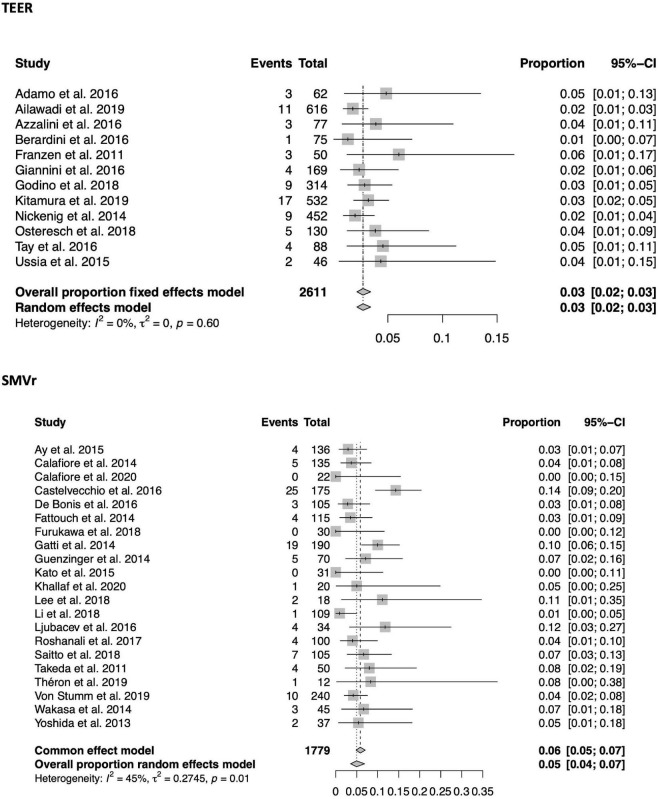
Forrest plots of the comparison between TEER and SMVr for in-hospital mortality. TEER, transcatheter edge-to-edge repair; SMVr, surgical mitral valve repair.

**FIGURE 4:**
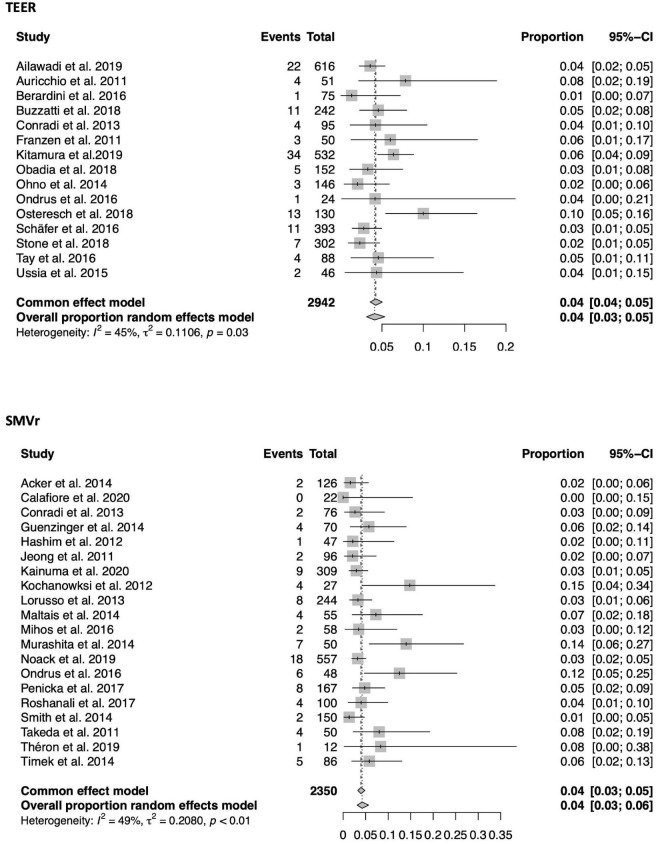
Forrest plots of the comparison between TEER and SMVr for 30-day mortality. TEER, transcatheter edge-to-edge repair; SMVr, surgical mitral valve repair.

A total of 33 of the 56 included studies (21 SMVr, 12 TEER) reported in-hospital mortality, which was significantly lower in the TEER compared to the SMVr cohort [3% (95%-CI 0.02–0.03, *I*^2^ = 0%) vs. 5% (95%-CI 0.04–0.07, *I*^2^ = 45%); see also Figure 3]. 30-day mortality was reported in 35 of the included studies (15 TEER, 20 SMVr) and comparable between both groups [4% in TEER (95%-CI 0.03–0.05, *I*^2^ = 45%) vs. 4% in SMVr (95%-CI 0.03–0.06, *I*^2^ = 49%); see also Figure 4].

**FIGURE 5:**
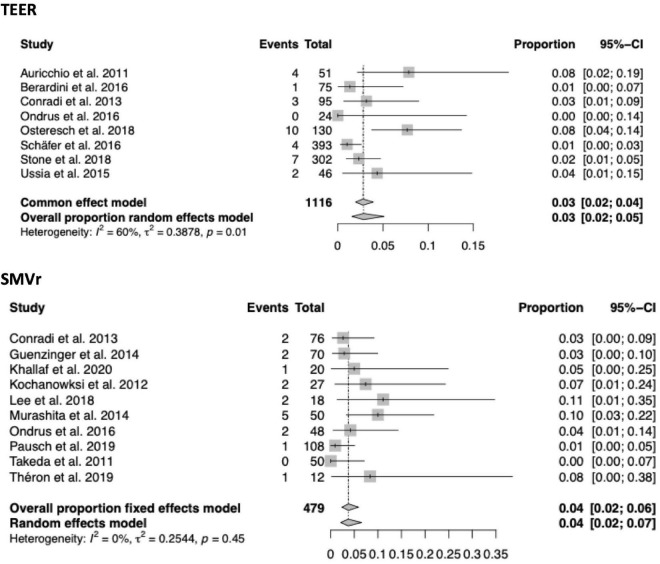
Forrest plots of the comparison between TEER and SMVr for 30-day cardiac death. TEER, transcatheter edge-to-edge repair; SMVr, surgical mitral valve repair.

A total of 8 TEER and 10 SMVr studies explicitly reported 30-day cardiac death, which was comparable between both treatment strategies [3% (95%-CI 0.02–0.05, *I*^2^ = 60%) vs. 4% (95%-CI 0.02–0.06, *I*^2^ = 0%); see also Figure 5]. Regarding in-hospital and long-term mortality cardiac death was not analyzed due to limited data.

### One-year outcomes

**FIGURE 6:**
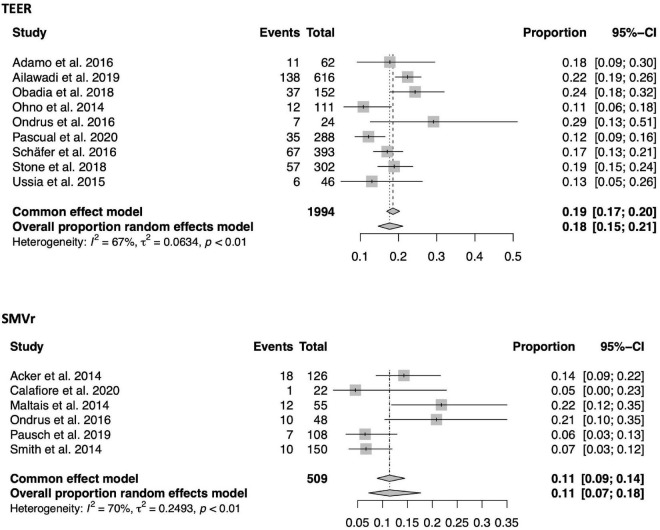
Forrest plots of the comparison between TEER and SMVr for 1-year mortality. TEER, transcatheter edge-to-edge repair; SMVr, surgical mitral valve repair.

A total of 9 TEER and 6 SMVr studies containing 1,994 and 509 patients separately reported on 1-year mortality (Figure 6). 1-year mortality was 18% in the TEER and 11% in the SMVr cohort [(95%-CI 0.15–0.21, *I*^2^ = 67%) vs. (0.07–0.18, *I*^2^ = 70%)]. As the 95%-CI of the TEER and SMVr groups did overlap by a small margin, a tendency toward lower 1-year mortality in SMVr was observed, however, this finding lacks statistical significance (see also Figure 2).

### Mitral regurgitation reduction and symptomatic benefit

**FIGURE 7:**
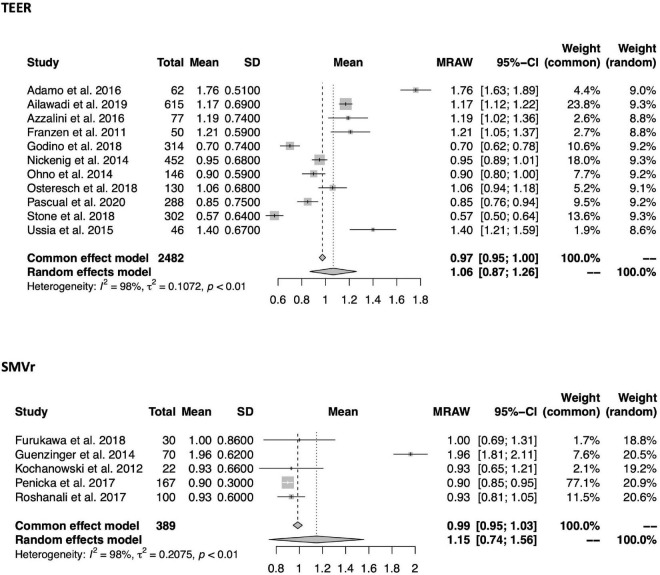
Forrest plots of the comparison between TEER and SMVr for NYHA reduction. Mean NYHA reduction grade with pooled standard deviation were calculated for each study and included in the model. TEER, transcatheter edge-to-edge repair; SMVr, surgical mitral valve repair.

**FIGURE 8:**
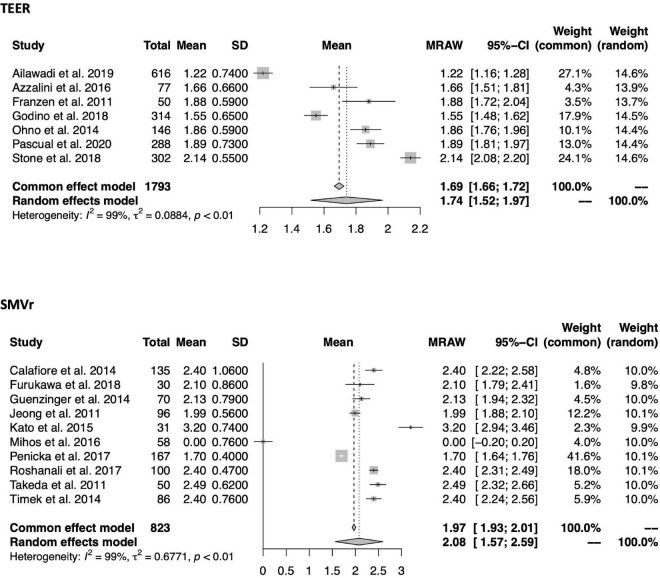
Forrest plots of the comparison between TEER and SMVr for MR grade reduction. Mean MR reduction grade with pooled standard deviation were calculated for each study and included in the model. TEER, transcatheter edge-to-edge repair; SMVr, surgical mitral valve repair; MR: Mitral regurgitation.

Within a mean follow-up period of 13.3 ± 4.4 months in TEER and 20.2 ± 22.9 months in SMVr, NYHA class reduction was comparable between both groups [1.06 (95%-CI 0.87–1.26, *I*^2^ = 98%) vs. 1.15 (0.74–1.56, *I*^2^ = 98%); see also Figure 7]. Likewise, there was no difference in MR grade reduction within a mean follow-up period of 13.9 ± 5.1 months in TEER and 26.9 ± 23.1 months in SMVr [1.74 (95%-CI 1.52–1.97, *I*^2^ = 99%) vs. 2.08 (1.57–2.59, *I*^2^ = 99%); see also Figures 2, 8].

The reported necessity of reoperation or reintervention within a mean follow-up period of 18.1 ± 14.2 months in TEER and 55.7 ± 28.3 months in SMVr did not differ significantly and was comparably low in both cohorts [3% (95%-CI 0.02–0.05, *I*^2^ = 68%) vs. 3% (0.01–0.05, *I*^2^ = 76%); see also Supplementary Figure 2].

### Meta-regression analysis

Meta-regression analysis of 30-day mortality was performed for all potential variables if at least 10 studies individually reported on the variable of interest according to the Cochrane Handbook for Systematic Reviews of Interventions (27).

Baseline NYHA class remained a significant correlate of 30-day mortality. In the TEER cohort, NYHA IV (estimate: 3.1, 95%-CI 1.33–4.93, *p* < 0.001) and in the SMVr cohort, NYHA III + IV (estimate: 2.4, 95%-CI 0.82–4.04; *p* = 0.003) presented as a significant moderator of 30-day mortality (Table 2).

**TABLE 2:** Meta-regression of potential moderators in the TEER and the SMVr cohort for 30-day mortality.

Moderator	Estimate	95%-CI	*P*-value
***TEER***			
LVEF%	−0.0205	−0.0701–0.0291	0.42
Age	0.0761	−0.0869–0.2390	0.36
Male	1.2145	−2.3404–4.7694	0.50
Atrial fibrillation	−0.3608	−2.9138–2.1922	0.78
NYHA III or IV	−1.4099	−5.3404–2.5207	0.48
NYHA IV	3.1285	1.3270–4.9300	**<0**.**001**
Diabetes	−1.7856	−6.7182–3.1470	0.48
Previous PCI	−2.8793	−6.9456–1.1869	0.17
MR grade	−0.2865	−1.3498–0.7768	0.60
COPD/lung disease	0.3722	−2.1146–2.8591	0.77
***SMVr***			
LVEF	−0.0297	−0.0815–0.0222	0.26
Age	0.0209	−0.0375–0.0793	0.48
Men	0.5603	−2.7204–3.8410	0.74
Atrial fibrillation	1.3738	−0.6692–3.4167	0.19
NYHA III or IV	2.4251	0.8149–4.0352	**0**.**003**
Diabetes	−0.7116	−3.1620 1.7387	0.57
MR grade	−0.0759	−0.6929–0.5412	0.81

COPD, chronic obstructive pulmonary disease; LVEF, left ventricular ejection fraction; MR, mitral regurgitation; PCI, percutaneous coronary intervention, NYHA, New York heart association class; SMVr, surgical mitral valve repair; TEER, transcatheter edge-to-edge repair. Significant *p*-values are presented in bold.

## Discussion

To the best of our knowledge, by gathering data from 8,287 patients with FMR this analysis represents the largest study comparing TEER and SMVr so far. The main findings can be summarized as follows:

• TEER patients were significantly older, presented with a significantly higher comorbidity burden, poorer LVEF and higher operative risk.
• Despite this high-risk profile, in-hospital mortality was significantly lower in the TEER cohort (3 vs. 5%).
• A comparable 30-day mortality rate was observed in both groups (4%).
• In the younger and substantially less fragile SMVr cohort, a non-significant tendency toward lower 1-year mortality was found (11 vs. 18%).
• Long-term MR and NYHA class reduction were comparable between both treatment strategies.

This meta-analytic approach compares TEER using the MC system and SMVr by calculated estimation of event rates for each treatment. Unlike standard meta-analysis, the meta-analytic approach is not limited to studies directly comparing TEER and SMVr for treatment of FMR. This enables the inclusion of studies reporting on both DMR and FMR or single-arm studies reporting on SMVr or TEER procedures only. Therefore, precise event estimators with narrow 95%-CIs can be derived from an even larger patient collective.

Reported in-hospital mortality of FMR patients undergoing TEER procedures ranges between 1.8 and 6% and tend to decrease with growing experience and technical improvement (17, 28). In our study, data of FMR cohorts treated over a period of 10 years were merged resulting in a real-world TEER collective with an in-hospital mortality estimator of 3% (95%-CI 0.02–0.03; *I*^2^ = 0%). This was significantly lower compared to the 5% in-hospital mortality estimator of SMVr despite significantly higher age and comorbidity burden, poorer LVEF and a twofold higher logistic EuroSCORE.

It has to be mentioned that MV surgery often comprises different treatment strategies like sternotomy or minimally invasive access, additional subvalvular treatment or ventricular remodeling such as chordal cutting/replacement (21, 23), edge-to-edge repair (29) or papillary muscle approximation (20, 24). Moreover, concomitant procedures like coronary artery bypass graft, tricuspid valve repair, Maze procedures or pulmonary vein isolation and left atrial appendage occlusion are frequently performed. In contrast, TEER avoids the cumulative risk of several procedures being performed at once and follows a highly standardized workflow resulting in minimized invasiveness and periprocedural risk. Concomitant cardiac pathologies, however, can be treated subsequently by staged treatment strategies of low-risk procedures such as catheter ablation (0.15%) (30), elective percutaneous coronary intervention (0.1%) (31) or percutaneous left atrial appendage occlusion (0.3%) (32).

An identical 4% rate of 30-day mortality for both cohorts seems notable considering the high-risk profile of TEER patients and additionally confirms its low-risk character. Meta-regression found baseline NYHA IV to be a significant modifier of 30-day mortality in the TEER cohort and baseline NYHA III and IV in the SMVr cohort. This is in accordance with previous findings indicating higher peri-operative risk and increased mortality in patients with advanced stages of heart failure (33). However, the COAPT trial demonstrated that TEER is safe and reduces mortality in patients with NYHA class IV, thus properly addressing this issue (34).

1-year mortality was 18% (95%-CI 0.15–0.21) in the TEER cohort showing a trend toward poorer outcome compared to 11% in the SMVr cohort (95%-CI 0.07–0.18). However, differences in age and comorbidity burden naturally contribute to these findings, which are in line with COAPT reporting 19.1% and lower than the FMR population of the EVEREST II trial with 22.4% and patients in MITRA-FR with 24.3% (8, 9, 17). Notably, this also reflects the evolution across more than one decade of TEER as well as the progress in patient selection.

NYHA and MR reduction as well as reoperation or reintervention rates were similar with both treatment strategies and demonstrate their efficacy. Shorter follow-up periods in the TEER cohort may impede comparability, however, 1-year results of TEER were repeatedly shown to be stable in the long term in randomized controlled trials (8, 9). Moreover, the included studies predominantly used first and second generation MC and substantial technical developments over the last few years further improved treatment results and durability as preliminary data from the EXPAND studies indicate (35). Similar results were achieved in the substantially fewer investigations on the novel PASCAL^®^ system in exclusively non-randomized trials mostly reporting on combined FMR and DMR collectives (13). Completion of the randomized comparison of MC and PASACAL^®^ in FMR patients within the CLASP IIF trial is expected in 2023/24 (36).

Regarding MV surgery in FMR, several studies have shown higher recurrence rates for MV repair opposed to an excellent long-term correction with MV replacement. However, MV replacement was associated with higher perioperative mortality and surgical repair still is recommended whenever applicable (12). Eventually, first results regarding interventional MV replacement in patients not suitable for TEER indicate a promising combination of a low-risk procedure and effective MR correction (37, 38).

Evidence favoring TEER for treatment of patients with FMR is constantly growing, however, there still is an ongoing debate about the optimal treatment strategy. This study adds a meta-analytic approach to the existing evidence comparing TEER and SMVr, currently the two most relevant treatment strategies. Results of a direct head-to-head comparison of both therapies are still not available. In this regard, completion of randomized controlled trials like the MATTERHORN study is eagerly expected (39).

### Limitations

One limitation of the meta-analytic approach is the comparison of patients included in studies, which did not primarily compare SMVr and TEER. Consequently, the presented estimators are not adjusted and risk-of-bias-assessment was not feasible due to the use of single-arm studies. However, this can also be considered a strength resulting in large datasets of patients undergoing TEER or SMVr. Additionally, adjustment for baseline characteristics might presumably be in favor of TEER. Since some variables are reported by a limited study number, meta-regression was not performed for all variables. Potential moderating influence of variables not examined by meta-regression analysis cannot be ruled out. Our studies’ endpoint is restricted to 1 year because many studies report different long-term follow-up periods impeding interpretation. Likewise, follow-up periods of the reported post-interventional NYHA and MR grades substantially differed between TEER and SMVr, which might affect comparability. NYHA and MR grade reduction were treated as numeric variables because many studies reported mean with standard deviation only, which necessitated a general comparison of means (22, 23, 40). Eventually, the fact that only studies reporting on first and second generation MC devices were included in this investigation limits its value. However, latest results of the novel PASCAL^®^ system as well as recent MC device generations would presumably emphasize the benefits of TEER.

## Conclusion

In this meta-analytic approach comprising >8,000 FMR patients treated with TEER or SMVr, TEER is associated with significantly lower in-hospital mortality, despite considerably higher age, comorbidity burden, operative risk, and poorer LVEF. This high-risk collective treated with TEER showed a non-significant tendency toward increased 1-year mortality. In terms of 30-day mortality as well as NYHA and MR grade reduction, comparable results were achieved with both treatment strategies.

The authors have no relationships relevant to the content of this paper to disclose. This research did not receive external funding.

### Impact on daily practice

Evidence regarding favorable treatment of FMR using TEER is constantly growing, whereas surgical MV repair and replacement have repeatedly shown moderate outcomes in this high-risk population. However, there still is an ongoing debate about the optimal treatment strategy due to a lacking direct comparison. With this meta-analytic approach, we were able to show similar mid- and long-term prognostic and symptomatic outcomes of TEER compared to SMVr in >8,000 FMR patients, despite an unfavorable baseline risk-profile of TEER patients. Results of a direct head-to-head comparison of both therapies are eagerly awaited upon completion of the randomized controlled MATTERHORN trial.

## Data availability statement

The raw data supporting the conclusions of this article will be made available by the authors, without undue reservation.

## Author contributions

DF, LS, and SM: conceptualization. BM, DF, MK, and RF: methodology. BM and DF: software and visualization. WR, LS, SM, MT, and BM: validation. BM, MP, and DF: formal analysis. RF, MP, and DF: investigation. RF, LS, and DF: data curation. DF, LS, and RF: writing—original draft preparation. LS, WR, and TS: writing—review and editing. SM, WR, TD, and MK: supervision. All authors contributed to the article and approved the submitted version.
