# Supplementary material for: Comparison of transcatheter edge-to-edge and surgical repair in patients with functional mitral regurgitation using a meta-analytic approach
Source: Front Cardiovasc Med. 2023 Jan 25;9:1063070. doi: 10.3389/fcvm.2022.1063070 (PMC9905105; doi:10.3389/fcvm.2022.1063070)
Supplement: Supplementary file 1 [file Data_Sheet_1.docx]

Supplemental Appendix

Page 2……S1 Literature search strategy

Page 3……S2 Forrest plots of the comparison between MitraClip and surgical mitral valve repair for 1-year reoperation or reintervention rates.

Page 4……S3 NIH rating of the included studies

1. Exp Mitral Valve Insufficiency
2. Exp Ventricular Dysfunction, Left
3. Functional mitral regurgitation.mp
4. Ischemic mitral regurgitation.mp
5. Heart Valve Prothesis Implantation/ or Cardiac Surgical Procedures/ or mitral vlave repair.mp or Mitral Vlave Annuloplasty/
6. Cardiac Catheterization/ or MitraClip.mp
7. Comparartive Study / or Multicenter Study/ or Clinical Study/ or “Proof of Concept Study”/ or Study.mp. or Observational Study/
8. Exp Myocardial Ischemia/
9. 2 or 3 or 4 or 8
10. 5 or 6
11. 7 and 11
12. Secondary mitral regurgitation.mp
13. 2 or 3 or 4 or 8 or 13
14. 1 and 7 and 10 and 14
15. limit 14 to yr="2010 - 2020"

**Figure S1** Literature search strategy

**TEER**

**SMVr**

**Figure S2** Forrest plots of the comparison between MitraClip and surgical mitral valve repair for 1-year reoperation or reintervention rates.

*TEER: Transcatheter edge-to-edge repair; SMVr: Surgical mitral valve repair*

| **Table S3** NIH rating of the included studies | |
| --- | --- |
| **Study** | **NIH** |
| Acker et al. 2014^1^ | good |
| Adamo et al. 2016^2^ | good |
| Ailawadi et al. 2019^3^ | good |
| Auricchio et al. 2011^4^ | fair |
| Ay et al. 2015^5^ | fair |
| Azzalini et al. 2016^6^ | good |
| Berardini et al. 2016^7^ | good |
| Buzzatti et al. 2018^8^ | good |
| Calafiore et al. 2014^9^ | good |
| Calafiore et al 2020^10^ | fair |
| Castelvecchio et al. 2016^11^ | good |
| Conradi et al. 2013^12^ | good |
| De Bonis et al. 2016^13^ | good |
| Fattouch et al. 2014^14^ | fair |
| Franzen et al. 2011^15^ | fair |
| Furukawa et al. 2018^16^ | fair |
| Gatti et al. 2014^17^ | fair |
| Giannini et al. 2016^18^ | good |
| Godino et al.2018^19^ | good |
| Guenzinger et al. 2014^20^ | fair |
| Hashim et al. 2012^21^ | poor |
| Jeong et al. 2011^22^ | good |
| Kainuma et al. 2020^23^ | good |
| Kato et al. 2015^24^ | good |
| Khallaf et al. 2020^25^ | good |
| Kitamura et al. 2019^26^ | good |
| Kochanowski et al. 2012^27^ | good |
| Lee et al. 2018^28^ | good |
| Li et al. 2018^29^ | good |
| Ljubacev et al. 2016^30^ | good |
| Lorusso et al. 2013^31^ | good |
| Maltais et al. 2014^32^ | good |
| Mihos et al. 2016^33^ | good |
| Murashita et al. 2014^34^ | good |
| Nickenig et al. 2014^35^ | good |
| Noack et al. 2019^36^ | good |
| Obadia et al. 2018^37^ | good |
| Ohno et al. 2014^38^ | good |
| Ondrus et al. 2016^39^ | fair |
| Osteresch et al. 2018^40^ | good |
| Pascual et al. 2020^41^ | good |
| Pausch et al. 2019^42^ | good |
| Penicka et al. 2017^43^ | good |
| Roshanali et al. 2017^44^ | good |
| Saitto et al. 2018^45^ | good |
| Schäfer et al. 2016^46^ | good |
| Smith et al. 2014^47^ | good |
| Stone et al. 2018^48^ | good |
| Takeda et al. 2011^49^ | good |
| Tay et al. 2016^50^ | good |
| Théron et al. 2019^51^ | good |
| Timek et al. 2014^52^ | fair |
| Ussia et al. 2015^53^ | good |
| Von Stumm et al. 2019^54^ | good |
| Wakasa et al. 2014^55^ | good |
| Yoshida et al. 2013^56^ | good |

1. Acker MA, Parides MK, Perrault LP, et al. Mitral-Valve Repair versus Replacement for Severe Ischemic Mitral Regurgitation. *New England Journal of Medicine*. 2014;370(1):23-32. doi:10.1056/NEJMoa1312808

2. Adamo M, Chiari E, Curello S, et al. Mitraclip therapy in patients with functional mitral regurgitation and missing leaflet coaptation: is it still an exclusion criterion? *European Journal of Heart Failure*. 2016;18(10):1278-1286. doi:10.1002/ejhf.520

3. Ailawadi G, Lim DS, Mack MJ, et al. One-Year Outcomes after MitraClip for Functional Mitral Regurgitation. *Circulation*. 2019;139(1):37-47. doi:10.1161/CIRCULATIONAHA.117.031733

4. Auricchio A, Schillinger W, Meyer S, et al. Correction of Mitral Regurgitation in Nonresponders to Cardiac Resynchronization Therapy by MitraClip Improves Symptoms and Promotes Reverse Remodeling. *J Am Coll Cardiol*. 2011;58(21):2183-2189. doi:10.1016/j.jacc.2011.06.061

5. Ay Y, Erkin A, Kara I, Aydin C, Ay NK, Zeybek R. Posterior leaflet segment 2 plication in ischemic mitral regurgitation repair. *Asian Cardiovascular and Thoracic Annals*. 2015;23(5):517-524. doi:10.1177/0218492314547088

6. Azzalini L, Millán X, Khan R, et al. Impact of left ventricular function on clinical outcomes of functional mitral regurgitation patients undergoing transcatheter mitral valve repair. *Catheterization and Cardiovascular Interventions*. 2016;88(7):1124-1133. doi:10.1002/ccd.26460

7. Berardini A, Biagini E, Saia F, et al. Percutaneous mitral valve repair: The last chance for symptoms improvement in advanced refractory chronic heart failure? *International Journal of Cardiology*. 2017;228:191-197. doi:10.1016/j.ijcard.2016.11.241

8. Buzzatti N, Denti P, Scarfò IS, et al. Mid-term outcomes (up to 5 years) of percutaneous edge-to-edge mitral repair in the real-world according to regurgitation mechanism: A single-center experience. *Catheterization and Cardiovascular Interventions*. 2019;94(3):427-435. doi:10.1002/ccd.28029

9. Calafiore AM, Iacò AL, Clemente D, et al. Repair or prosthesis insertion in ischemic mitral regurgitation: Two faces of the same medal. *IJC Heart and Vessels*. 2014;3:32-36. doi:10.1016/j.ijchv.2014.02.002

10. Calafiore AM, Totaro A, de Amicis V, et al. Surgical mitral plasticity for chronic ischemic mitral regurgitation. *Journal of Cardiac Surgery*. 2020;35(4):772-778. doi:10.1111/jocs.14487

11. Castelvecchio S, Parolari A, Garatti A, et al. Surgical ventricular restoration plus mitral valve repair in patients with ischaemic heart failure: risk factors for early and mid-term outcomes. *European Journal of Cardio-Thoracic Surgery*. 2016;49(4):e72-e79. doi:10.1093/ejcts/ezv478

12. Conradi L, Treede H, Rudolph V, et al. Surgical or percutaneous mitral valve repair for secondary mitral regurgitation: comparison of patient characteristics and clinical outcomes. *European Journal of Cardio-Thoracic Surgery*. 2013;44(3):490-496. doi:10.1093/ejcts/ezt036

13. de Bonis M, Lapenna E, Barili F, et al. Long-term results of mitral repair in patients with severe left ventricular dysfunction and secondary mitral regurgitation: Does the technique matter? *European Journal of Cardio-thoracic Surgery*. 2016;50(5):882-889. doi:10.1093/ejcts/ezw139

14. Fattouch K, Castrovinci S, Murana G, et al. Papillary muscle relocation and mitral annuloplasty in ischemic mitral valve regurgitation: Midterm results. *The Journal of Thoracic and Cardiovascular Surgery*. 2014;148(5):1947-1950. doi:10.1016/j.jtcvs.2014.02.047

15. Franzen O, van der Heyden J, Baldus S, et al. MitraClip® therapy in patients with end-stage systolic heart failure. *European Journal of Heart Failure*. 2011;13(5):569-576. doi:10.1093/eurjhf/hfr029

16. Furukawa K, Yano M, Nakamura E, Nishimura M, Nakamura K. Mid-term results of mitral valve repair for ischemic mitral regurgitation adjusted according to the degree of remodeling progression. *General Thoracic and Cardiovascular Surgery*. 2018;66(12):707-715. doi:10.1007/s11748-018-1000-4

17. Gatti G, Dell’Angela L, Pinamonti B, et al. Asymmetric ring annuloplasty for ischemic mitral regurgitation: early and mid-term outcomes. *J Heart Valve Dis*. 2014;23(6):695-706. http://www.ncbi.nlm.nih.gov/pubmed/25790616

18. Giannini C, Fiorelli F, Colombo A, et al. Right ventricular evaluation to improve survival outcome in patients with severe functional mitral regurgitation and advanced heart failure undergoing MitraClip therapy. *International Journal of Cardiology*. 2016;223:574-580. doi:10.1016/j.ijcard.2016.08.189

19. Godino C, Scotti A, Taramasso M, et al. Two-year cardiac mortality after MitraClip treatment of functional mitral regurgitation in ischemic and non-ischemic dilated cardiomyopathy. *International Journal of Cardiology*. 2018;269(2017):33-39. doi:10.1016/j.ijcard.2018.06.041

20. Guenzinger R, Schneider EP, Guenther T, et al. Three-dimensional valve repair - The better care? Midterm results of a saddle-shaped, rigid annuloplasty ring in patients with ischemic mitral regurgitation. *Journal of Thoracic and Cardiovascular Surgery*. 2014;148(1):176-182. doi:10.1016/j.jtcvs.2013.08.071

21. Hashim SW, Youssef SJ, Ayyash B, et al. Pseudoprolapse of the anterior leaflet in chronic ischemic mitral regurgitation: Identification and repair. *The Journal of Thoracic and Cardiovascular Surgery*. 2012;143(4):S33-S37. doi:10.1016/j.jtcvs.2011.09.063

22. Jeong DS, Lee HY, Kim WS, et al. Long-term echocardiographic follow-up after posterior mitral annuloplasty using a vascular strip for ischemic mitral regurgitation Ten-years of experience at a single center. *Journal of Korean Medical Science*. 2011;26(12):1582-1590. doi:10.3346/jkms.2011.26.12.1582

23. Kainuma S, Toda K, Miyagawa S, et al. Restrictive mitral annuloplasty with or without coronary artery bypass grafting in ischemic mitral regurgitation. *ESC Heart Failure*. 2020;7(4):1560-1570. doi:10.1002/ehf2.12705

24. Kato Y, Bando K, Fukui T, Mahara K, Takanashi S. Surgical Treatment of Functional Mitral Regurgitation Involving the Subvalvular Apparatus. *Journal of Cardiac Surgery*. 2015;30(1):27-34. doi:10.1111/jocs.12459

25. Khallaf A, Elzayadi M, Alkady H, Elnaggar A. Results of surgical coronary revascularization alone versus combined surgical revascularization and mitral valve repair in patients with moderate ischemic mitral regurgitation. *Heart Surgery Forum*. 2020;23(3):E270-E275. doi:10.1532/hsf.2773

26. Kitamura M, Kaneko H, Schlüter M, et al. Predictors of mortality in ischaemic versus non-ischaemic functional mitral regurgitation after successful transcatheter mitral valve repair using MitraClip: results from two high-volume centres. *Clinical Research in Cardiology*. 2019;108(3):264-272. doi:10.1007/s00392-018-1352-x

27. Kochanowski J, Piątkowski R, Grabowski M, et al. Utility of stress echocardiography in selecting the optimal mitral valve procedure in patients with severe ischemic mitral regurgitation undergoing coronary artery bypass grafting. *Polish Archives of Internal Medicine*. 2012;122(5):217-225. doi:10.20452/pamw.1218

28. Lee ML, Chen TH, Huang H da, Hou SM. Mitral valve repair versus replacement in patients with ischemic mitral regurgitation. *Journal of Thoracic Disease*. 2018;10(5):2820-2828. doi:10.21037/jtd.2018.04.93

29. Li B, Chen S, Sun H, et al. Mitral valve annuloplasty versus replacement for severe ischemic mitral regurgitation. *Scientific Reports*. 2018;8(1). doi:10.1038/s41598-018-19909-7

30. Ljubačev A, Medved I, Oštrí M, Zuvić-Butorac M, Sokolić J. Mitral regurgitation and coronary artery bypass surgery : Comparison of mitral valve repair and replacement. *Acta Chirurgica Belgica*. 2013;113(3):187-191. doi:10.1080/00015458.2013.11680909

31. Lorusso R, Gelsomino S, Vizzardi E, et al. Mitral valve repair or replacement for ischemic mitral regurgitation? the Italian Study on the Treatment of Ischemic Mitral Regurgitation (ISTIMIR). *Journal of Thoracic and Cardiovascular Surgery*. 2013;145(1):128-139. doi:10.1016/j.jtcvs.2012.09.042

32. Maltais S, Tchantchaleishvili V, Schaff H v., et al. Management of severe ischemic cardiomyopathy: Left ventricular assist device as destination therapy versus conventional bypass and mitral valve surgery. *Journal of Thoracic and Cardiovascular Surgery*. 2014;147(4):1246-1250. doi:10.1016/j.jtcvs.2013.04.012

33. Mihos CG, Capoulade R, Yucel E, Melnitchouk S, Hung J. Combined papillary muscle sling and ring annuloplasty for moderate-to-severe secondary mitral regurgitation. *Journal of Cardiac Surgery*. 2016;31(11):664-671. doi:10.1111/jocs.12843

34. Murashita T, Okada Y, Kanemitsu H, et al. Midterm outcomes of chordal cutting in combination with downsized ring annuloplasty for ischemic mitral regurgitation. *Annals of Thoracic and Cardiovascular Surgery*. 2014;20(6):1008-1015. doi:10.5761/atcs.oa.13-00293

35. Nickenig G, Estevez-Loureiro R, Franzen O, et al. Percutaneous mitral valve edge-to-edge Repair: In-hospital results and 1-year follow-up of 628 patients of the 2011-2012 pilot European Sentinel Registry. *J Am Coll Cardiol*. 2014;64(9):875-884. doi:10.1016/j.jacc.2014.06.1166

36. Noack T, Cuartas MM, Kiefer P, et al. Isolated mitral valve repair in patients with reduced left ventricular ejection fraction. *Annals of Thoracic and Cardiovascular Surgery*. 2019;25(6):326-335. doi:10.5761/atcs.oa.19-00093

37. Obadia JF, Messika-Zeitoun D, Leurent G, et al. Percutaneous Repair or Medical Treatment for Secondary Mitral Regurgitation. *New England Journal of Medicine*. 2018;379(24):2297-2306. doi:10.1056/nejmoa1805374

38. Ohno Y, Attizzani GF, Capodanno D, et al. Association of tricuspid regurgitation with clinical and echocardiographic outcomes after percutaneous mitral valve repair with the MitraClip System: 30-day and 12-month follow-up from the GRASP Registry. *European Heart Journal Cardiovascular Imaging*. 2014;15(11):1246-1255. doi:10.1093/ehjci/jeu114

39. Ondrus T, Bartunek J, Vanderheyden M, et al. Minimally invasive mitral valve repair for functional mitral regurgitation in severe heart failure: MitraClip versus minimally invasive surgical approach. *Interactive Cardiovascular and Thoracic Surgery*. 2016;23(5):784-789. doi:10.1093/icvts/ivw215

40. Osteresch R, Diehl K, Kühl M, et al. Impact of right heart function on outcome in patients with functional mitral regurgitation and chronic heart failure undergoing percutaneous edge-to-edge-repair. *Journal of Interventional Cardiology*. 2018;31(6):916-924. doi:10.1111/joic.12566

41. Pascual I, Carrasco-Chinchilla F, Benito-Gonzalez T, et al. Transcatheter Mitral Repair for Functional Mitral Regurgitation According to Left Ventricular Function: A Real-Life Propensity-Score Matched Study. *Journal of Clinical Medicine*. 2020;9(6):1792. doi:10.3390/jcm9061792

42. Pausch J, Harmel E, Sinning C, Reichenspurner H, Girdauskas E. Standardized subannular repair for type IIIb functional mitral regurgitation in a minimally invasive mitral valve surgery setting†. *European Journal of Cardio-Thoracic Surgery*. 2019;56(5):968-975. doi:10.1093/ejcts/ezz114

43. Penicka M, Kotrc M, Ondrus T, et al. Minimally invasive mitral valve annuloplasty confers a long-term survival benefit compared with state-of-the-art treatment in heart failure with functional mitral regurgitation. *International Journal of Cardiology*. 2017;244:235-241. doi:10.1016/j.ijcard.2017.06.029

44. Roshanali F, Vedadian A, Shoar S, Naderan M, Mandegar MH. Efficacy of papillary muscle approximation in preventing functional mitral regurgitation recurrence in high-risk patients with ischaemic cardiomyopathy and mitral regurgitation. *Acta Cardiologica*. 2013;68(3):271-278. doi:10.1080/ac.68.3.2983421

45. Saitto G, Lio A, Russo M, et al. Mitral Valve Repair with a Semi-Rigid C-Band Annuloplasty Ring in Ischemic Mitral Regurgitation: Still a Viable Surgical Option? *J Heart Valve Dis*. 2018;27(1):47-54.

46. Schäfer U, Maisano F, Butter C, et al. Impact of Preprocedural Left Ventricular Ejection Fraction on 1-Year Outcomes After MitraClip Implantation (from the ACCESS-EU Phase I, a Prospective, Multicenter, Nonrandomized Postapproval Study of the MitraClip Therapy in Europe). *American Journal of Cardiology*. 2016;118(6):873-880. doi:10.1016/j.amjcard.2016.06.036

47. Smith PK, Puskas JD, Ascheim DD, et al. Surgical Treatment of Moderate Ischemic Mitral Regurgitation. *New England Journal of Medicine*. 2014;371(23):2178-2188. doi:10.1056/NEJMoa1410490

48. Stone GW, Lindenfeld J, Abraham WT, et al. Transcatheter Mitral-Valve Repair in Patients with Heart Failure. *New England Journal of Medicine*. 2018;379(24):2307-2318. doi:10.1056/nejmoa1806640

49. Takeda K, Sakaguchi T, Miyagawa S, et al. The extent of early left ventricular reverse remodelling is related to midterm outcomes after restrictive mitral annuloplasty in patients with non-ischaemic dilated cardiomyopathy and functional mitral regurgitation. *European Journal of Cardio-thoracic Surgery*. 2012;41(3):506-511. doi:10.1093/ejcts/ezr004

50. Tay E, Muda N, Yap J, et al. The MitraClip Asia-Pacific registry: Differences in outcomes between functional and degenerative mitral regurgitation. *Catheterization and Cardiovascular Interventions*. 2016;87(7):E275-E281. doi:10.1002/ccd.26289

51. Théron A, Morera P, Resseguier N, et al. Long-term results of surgical treatment of secondary severe mitral regurgitation in patients with end-stage heart failure: Advantage of prosthesis insertion. *Archives of Cardiovascular Diseases*. 2019;112(2):95-103. doi:10.1016/j.acvd.2018.09.006

52. Timek TA, Hooker RL, Collingwood R, et al. Five-year real world outcomes of GeoForm ring implantation in patients with ischemic mitral regurgitation. In: *Journal of Thoracic and Cardiovascular Surgery*. Vol 148. Mosby Inc.; 2014:1951-1956. doi:10.1016/j.jtcvs.2014.02.051

53. Ussia GP, Cammalleri V, Mazzotta E, et al. Cardiovascular medicine heart failure (CVM-HF) index as prognostic model for candidates to MitraClip therapy. *Heart and Vessels*. 2016;31(10):1633-1642. doi:10.1007/s00380-015-0772-z

54. von Stumm M, Dudde F, Gasser S, et al. Prognostic value of mitral valve tenting area in patients with functional mitral regurgitation. *Interactive Cardiovascular and Thoracic Surgery*. 2020;30(3):431-438. doi:10.1093/icvts/ivz291

55. Wakasa S, Kubota S, Shingu Y, Ooka T, Tachibana T, Matsui Y. The extent of papillary muscle approximation affects mortality and durability of mitral valve repair for ischemic mitral regurgitation. *Journal of Cardiothoracic Surgery*. 2014;9(1). doi:10.1186/1749-8090-9-98

56. Yoshida K, Okada K, Miyahara S, et al. Mitral valve replacement versus annuloplasty for treating severe functional mitral regurgitation. *General Thoracic and Cardiovascular Surgery*. 2014;62(1):38-47. doi:10.1007/s11748-013-0297-2
